# BA.5 and Monkeypox: A wakeup call for public health in Pakistan

**DOI:** 10.1016/j.amsu.2022.104413

**Published:** 2022-08-17

**Authors:** Muhammad Bilal Islam, Shizra Jawed, Muhammad Hamayl Zeeshan

**Affiliations:** Department of Internal Medicine, Dow Medical College, Dow University of Health Sciences, Karachi, Pakistan

Dear Editor,

Since its appearance (April 2022), the BA.5 lineage of the omicron variant has begun to dominate the world. According to the available data, 54 countries, including the United States and European countries, have reported outbreaks of the BA.5 variant with 11% global prevalence. Interestingly, BA.5.1 has risen in proportion, accounting for 80% of sequences detected only in European countries [1]. The rapid growth rate of this variant may be due to its ability to evade immune protection acquired through the previous infection and suggests to be more transmissible than the other lineages [2]. Moreover, Pakistan has reported cases associated with the BA.5 variant which is raising several concerns about the government's ability to tackle the health care dilemma [3].

Amidst the Covid-19 still shadowing us, there has been an outbreak of another virus known as Monkeypox which is a viral zoonosis that belongs to the Orthopoxvirus genus of the Poxviridae family containing double-stranded DNA. Since May 2022, monkeypox cases have been reported from both countries where it's widespread and those that are not [4]. So far, 21,148 confirmed cases of monkeypox are present, in which 20,080 cases were reported in countries that were previously not exposed to Monkeypox [5]. After an incubation period of usually one to two weeks, the disease usually begins with fever, fatigue, muscle soreness, and other flu-like symptoms that lead to a rash. Usually diagnosed via a blood sample, health care workers may also take a tissue sample from an open sore lesion for PCR testing [6]. Monkeypox spread follows the same human-to-human transmission that includes intimate contact, respiratory secretions, or even direct contact with a monkeypox rash. The World Health Organization (WHO) has stated monkeypox virus as a worldwide health crisis, and this classification is the highest alert that the WHO can issue following a worldwide upsurge in cases [7].

If the monkeypox virus, along with the B.A.5 omicron variant, looms over Pakistan, it can create havoc and panic in the country with a deleterious impact on the already worsening public health care. COVID-19 has exposed the vulnerability of betting on trade and disregarding investment, with almost 4.64% expected loss in the GDP of Pakistan [8]. This emphasizes that Pakistan would not be economically capable of sustaining another world health emergency, let alone two simultaneously. Pakistan has been very reluctant to improve its public health infrastructure and facilities, and this lacking was shown clearly with poor or almost no proper management during the peak of the Covid 19 pandemic. The citizens did not abide by the Standard Operating Procedures (SOPs) put forth till very late, and no accountability was done even when vaccination was made compulsory.

A crucial lesson gleaned from the detrimental COVID-19 pandemic is that the interconnectedness of the current world makes the threat of any of these viral infections real and imminent. Therefore, it is imperative for the government to put in place a system that diligently and rigorously tracks the prevalence of infectious diseases in Pakistan, consequently leading to the implementation of specific and effective policies. Given the substandard health system of Pakistan, it is more vulnerable to outbreaks hence early preventive measures are essential. Strict adherence to standard operating procedures (SOPs), early detection, and vaccinations play a vital role in hindering virus transmissions. According to a study, there was a worldwide decrease in 63% of total death after the first year of covid vaccination which emphasizes the need for efficient vaccine administration [9] Additionally, Awareness of the signs and symptoms among the locals needs to be reinforced for timely diagnosis and treatment. To conclude, viral outbreaks are on the rise, and if their spread is not curtailed by taking bold steps and consistent management choices, it may result in an unprecedented public health crisis.

## Ethical approval

Not Required.

## Sources of funding

No Funding.

## Author contribution

Muhammad Bilal Islam: Conception of the study, drafting of the work, final approval, and agreeing to the accuracy of the work.

Shizra Jawed: Drafting of the work, final approval and agreeing to the accuracy of the work.

Muhammad Hamayl Zeeshan: Supervision and critical revision of the manuscript, editing, final approval, and agreeing to the accuracy of the work.

## Research registration number

1. Name of the registry:

2. Unique Identifying number or registration ID:

3. Hyperlink to your specific registration (must be publicly accessible and will be checked):

## Guarantor

Muhammad Bilal Islam.

Shizra Jawed.

Muhammad Hamayl Zeeshan.

## Declaration of competing interest

None.
